# Supplementary material for: Brain microdialysis to assess trace elements dynamics in traumatic brain injury: An exploratory study
Source: PLoS One. 2025 Jun 16;20(6):e0326023. doi: 10.1371/journal.pone.0326023 (PMC12169518; doi:10.1371/journal.pone.0326023)
Supplement: S1 Table — Abbreviations: LOD: detection limit; LOQ: quantification limit; CVr: repetability; CVR: reproductibility. (DOCX) [file pone.0326023.s001.docx]

**S1 Table.** Analytical parameters of the internal quality controls (ClinCheck Controls, Recipe) used to validate our methods in two matrices (serum and urine).

| **Trace Element** | **LOD (µg/L)** | **LOQ (µg/L)** | **CVr (%)** | **CVR (%)** |
| --- | --- | --- | --- | --- |
| Chromium (Cr) | 0.189 | 0.559 | 3.30% | 4.50% |
| Manganese (Mn) | 0.123 | 0.377 | 3.90% | 7.90% |
| Cobalt (Co) | 0.003 | 0.015 | 1.80% | 4.50% |
| Copper (Cu) | 1.147 | 3.691 | 1.70% | 3.10% |
| Zinc (Zn) | 0.421 | 5.536 | 1.20% | 3.90% |
| Selenium (Se) | 0.614 | 1.680 | 1.40% | 2.50% |
| Molybdenum (Mo) | 0.023 | 0.074 | 2.00% | 2.80% |

LOD : detection limit ; LOQ : quantification limit ; CVr : repetability ; CVR : reproductibility
